# Correction: Impact of ligand fields on Kubas interaction of open copper sites in MOFs with hydrogen molecules: an electronic structural insight

**DOI:** 10.1039/d4ra90119c

**Published:** 2024-10-15

**Authors:** Trang Thuy Nguyen, Hoan Van Tran, Linh Hoang Nguyen, Hoang Minh Nguyen, Thang Bach Phan, Toan Nguyen-The, Yoshiyuki Kawazoe

**Affiliations:** a Faculty of Physics, University of Science, Vietnam National University Hanoi Vietnam nguyenthuytrang@hus.edu.vn; b Key Laboratory for Multiscale Simulation of Complex Systems, University of Science, Vietnam National University Hanoi Vietnam; c School of Engineering Physics, Hanoi University of Science and Technology Hanoi Vietnam; d Center for Innovative Materials and Architectures, Vietnam National University Ho Chi Minh City Vietnam; e Vietnam National University Ho Chi Minh City Vietnam; f Key Laboratory for Multiscale Simulation of Complex Systems, University of Science, Vietnam National University Hanoi Vietnam; g New Industry Creation Hatchery Center, Tohoku University Sendai 980-8579 Japan; h Department of Physics and Nanotechnology, SRM Institute of Science and Technology Kattankulathur 603203 Tamilnadu India; i School of Physics, Institute of Science, Suranaree University of Technology 111 University Avenue Nakhon Ratchasima 30000 Thailand

## Abstract

Correction for ‘Impact of ligand fields on Kubas interaction of open copper sites in MOFs with hydrogen molecules: an electronic structural insight’ by Trang Thuy Nguyen *et al.*, *RSC Adv.*, 2024, **14**, 26611–26624, https://doi.org/10.1039/D4RA03946G.

The authors regret that the one of the affiliations (affiliation *c*) was incorrectly shown in the original manuscript. The corrected list of affiliations is as shown here.

The Royal Society of Chemistry apologises for these errors and any consequent inconvenience to authors and readers.

